# Evaluation of Effectiveness of Bio-Micro Injection in Treating Alopecia Universalis: A Non-Randomized Interventional Study: Mesotherapy in Treating Alopecia Universalis

**DOI:** 10.31661/gmj.v14i.3750

**Published:** 2025-08-08

**Authors:** Shahzad Shirzad, Mahdis Miladi

**Affiliations:** ^1^ Department of Dermatology, Tehran University of Medical Sciences, Tehran, Iran; ^2^ Department of Pediatrics, Tehran University of Medical Sciences, Tehran, Iran

**Keywords:** Alopecia Areata, Alopecia Universalis, Mesotherapy, JAK Inhibitor

## Abstract

**Background:**

Alopecia areata (AA) is an autoimmune disorder causing non-scarring hair loss, ranging from patchy baldness to total body hair loss (alopecia universalis, AU). AA significantly impacts health-related quality of life. While mesotherapy has shown potential as a localized therapy, its efficacy in AU remains understudied. This study aims to assess the therapeutic impact of mesotherapy in a substantial cohort of 2,000 patients diagnosed with alopecia universalis at Dr. Shirzad Clinic between 2020 and 2024, thereby providing valuable insights into its potential as a treatment modality.

**Materials and Methods:**

We conducted a non-randomized interventional study on 2,000 alopecia universalis patients across four treatment groups. Participants were divided into four treatment groups: one group received BMI alone, another received a combination of BMI and JAK inhibitors, the third group received only JAK inhibitors, and the fourth group was treated with a combination of BMI, JAK inhibitors, and adalimumab. All of these drugs were administered through mesotherapy techniques. They received the treatment once a month, for a year. Outcomes, including scalp hair regrowth and patient satisfaction, were assessed monthly over a year. Comorbidities were managed to prevent confounding effects.

**Results:**

A total of 2000 patients participated in this study, with an average age of 26.82 ±1.84 years. Treatment responses varied across groups, with children showing the highest success rates. In Group 1 (BMI only), 20% responded positively. Group 2 (BMI + JAK inhibitors) had a 30% had favorable outcomes. Group 3 (JAK inhibitors only) showed a 34% response rate. The combination treatment in Group 4 resulted in the highest success rate, with 42% of participants responding positively.

**Conclusion:**

This study highlights the unique contribution of mesotherapy, particularly when combined with systemic treatments such as BMI, JAK inhibitors, and adalimumab, as an effective and well-tolerated option for alopecia universalis. The large sample size and innovative application of mesotherapy enhance drug delivery, improve treatment outcomes, and reduce systemic side effects, underscoring its potential as a valuable treatment strategy for this challenging condition.

## Introduction

Alopecia areata (AA) is an autoimmune disorder characterized by patches of nonscarring hair loss. This disease shortens the hair growth phase (anagen), accelerates the resting phase (telogen), and ultimately results in hair loss [1]. AA affects around 1-2% of the population, with about 20% of cases having a familial history. Its severity varies from alopecia focalis (AF), involving circular patchy scalp hair loss, to total scalp hair loss (alopecia totalis) and complete body hair loss (alopecia universalis) [2]. AA has been found to affect quality of life as significantly as other chronic skin conditions, such as psoriasis and atopic dermatitis [3]. While alopecia areata (AA) is generally regarded as a non-life-threatening condition, it can significantly affect patients’ health-related quality of life (HRQoL) [4]. Research indicates that individuals with AA frequently endure considerable emotional and psychological challenges [5][6]. Both children and adults report experiencing social stigma, including harassment, exclusion, intrusive stares, and being mistakenly perceived as undergoing chemotherapy due to their hair loss. Numerous studies have highlighted a strong association between AA and psychological comorbidities such as anxiety and depression [7][8][9]. The cause of AA is believed to be multifactorial. The prevailing theory is that AA is an autoimmune disorder, where cytotoxic T cells target follicular autoantigens, leading to hair follicle destruction. This process also involves the loss of melanocytes within the follicles, which explains the regrowth of depigmented hairs alongside the preservation of unpigmented ones [10][11][12][13]. Genetic susceptibility, particularly involving TNF and IL-1 genes, contributes to disease development. Immunological studies have revealed increased levels of inflammatory cytokines like IL-12, IL-1, IL-6, IL-10, and IL-4 in affected tissues. The effectiveness of immunosuppressive and immunomodulatory therapies further supports the autoimmune nature of AA [14][15]. Although no special treatment is approved by Federal Drug Administration (FDA) for AA, various topical and systemic therapies have been tried with mixed results. Mild cases often resolve spontaneously within a year. The probability of spontaneous full hair regrowth in cases of alopecia totalis (AT) or alopecia universalis (AU) is below 10% [16]. Treatment options for AA are typically restricted to topical steroids, intralesional steroids, irritants, and minoxidil. For more extensive cases like alopecia totalis (AT) or alopecia universalis (AU), systemic therapies are often necessary due to the impracticality of topical treatments. AT and AU are associated with a worse prognosis and higher treatment failure rates compared to patchy alopecia areata (AA). In pediatric patients, common treatments include corticosteroids, irritants, sensitizers, and immunosuppressive agents. Recently, Janus kinase (JAK) inhibitors, particularly upadacitinib, have shown promise in treating both atopic dermatitis (AD) and AA [4][16][17]. Another therapeutic approach that has shown effectiveness for some patients with alopecia is mesotherapy [18]. Recent advancements have introduced Janus kinase (JAK) inhibitors, such as upadacitinib, which have shown promise in treating AA. Additionally, mesotherapy—a technique that involves the injection of small amounts of therapeutic agents directly into the skin—has emerged as a potential treatment modality. Introduced by Dr. Michel Pistor in 1976, mesotherapy alters drug absorption rates, allowing for localized therapeutic effects. While commonly used for cosmetic and pain management purposes, its application in treating alopecia is less conventional and requires further exploration. This study aims to address the gaps in existing research by evaluating the efficacy of mesotherapy in a large cohort of 2,000 patients diagnosed with alopecia universalis at Dr. Shirzad Clinic between 2020 and 2024. Specifically, we investigate the therapeutic effects of mesotherapy when combined with systemic treatments, including BMI, JAK inhibitors, and Adalimumab, to enhance drug delivery and minimize systemic side effects. By focusing on this innovative approach, we hope to provide valuable insights into effective treatment strategies for alopecia universalis.

## Methods and Materials

### Study Design and Population

We conducted a non-randomized interventional study to evaluate the effectiveness of mesotherapy in treating alopecia universalis, adhering to ethical guidelines with ethics code IR.TUMS.SPH.REC.1402.10.19 and IRCT code IRCT20241230056032N1. The non-randomized design was influenced by clinical judgment and patient preferences, as all participants were selected from a single dermatology specialty clinic. While this approach introduces potential bias, it reflects real-world clinical practice, where treatment decisions are often based on individual patient needs and circumstances.

### Participants and Inclusion Criteria

Eligible participants were those diagnosed with alopecia universalis, confirmed via biopsy. Individuals with a history of previous treatments, including corticosteroid therapies, were excluded based on predefined criteria. A total of 2,000 participants were enrolled, divided into four groups of 500 individuals, comprising men, women, and children aged between 5 and 60 years. Prior to treatment initiation, all participants underwent comprehensive blood testing, including complete blood count (CBC), thyroid function tests, and assessment of sex hormone levels. Participants with underlying health conditions, such as diabetes, thyroid disorders, polycystic ovary syndrome (PCOS), and iron-deficiency anemia, were managed concurrently to ensure these comorbidities did not interfere with study outcomes.

### Treatment Groups

Participants were assigned to one of four treatment groups based on the therapies they received:

1. Group 1: 176 children, 196 women, and 128 men treated solely with mesotherapy (BMI).

2. Group 2: 176 children, 195 women, and 129 men treated with a combination of mesotherapy and JAK inhibitors (tofacitinib and baricitinib).

3. Group 3: 176 children, 195 women, and 129 men treated exclusively with JAK inhibitors (tofacitinib and baricitinib).

4. Group 4: 177 children, 195 women, and 128 men treated with a combination of mesotherapy, JAK inhibitors, and adalimumab.

Mesotherapy sessions were conducted once a month at the treatment center. The rationale for selecting these specific combinations was based on emerging evidence suggesting that combining mesotherapy with systemic treatments could enhance therapeutic efficacy while minimizing systemic side effects.

## Outcome Measures

The primary outcomes were the initiation of hair growth on the scalp and patient satisfaction with hair regrowth, assessed through clinical examinations and validated patient-reported questionnaires. The validation process for these questionnaires involved ensuring reliability and consistency in measuring patient experiences and treatment outcomes.

### Limitations of Non-Randomization

The absence of a control group receiving a placebo or standard treatment is a notable limitation of this study, as it complicates the evaluation of the true efficacy of the interventions. Without a control group, it is challenging to account for the potential placebo effect, which can significantly influence patient-reported outcomes and perceived treatment success. The observed improvements in hair regrowth and patient satisfaction may be partially attributed to participants’ expectations and psychological factors associated with receiving treatment. To mitigate this concern, comprehensive baseline assessments, including blood tests and management of comorbidities, were employed to ensure that any observed effects were more likely due to the treatments administered rather than external factors. Additionally, the combination of clinical examinations and patient-reported questionnaires provided a more objective measure of treatment outcomes, although the lack of a control group limits the ability to definitively attribute improvements to the specific therapies used.

## Data Analysis

Data analysis involved several statistical methods to evaluate treatment effectiveness across different demographic groups. Descriptive statistics summarized key characteristics such as age, gender, and treatment outcomes within each group. Chi-square tests were utilized for categorical variables, such as gender, to compare response rates among different demographic categories. For continuous variables, t-tests assessed the correlation between age and treatment outcomes, providing insights into how age may influence efficacy. All statistical analyses were performed using Stata version 18, with a significance level set at p < 0.05. To address potential issues related to multiple comparisons, adjustments were made using methods such as the Bonferroni correction or the Holm-Bonferroni method, which help control the family-wise error rate. Missing data were excluded from the analysis, and results were presented as means ± standard deviation for continuous variables and frequencies for categorical variables, ensuring clarity in reporting findings. Overall, these statistical methods provided a robust framework for analyzing the data and drawing meaningful conclusions about treatment effectiveness across different groups.

### Ethical Consideration

The study protocol and informed consent form were reviewed and approved in accordance with ethical guidelines outlined in the Declaration of Helsinki [19]. Prior to enrollment, all participants received a comprehensive explanation of the study’s purpose, procedures, potential risks, and benefits. Written informed consent was then obtained from each participant, ensuring they fully understood their rights and had the opportunity to ask questions. For participants under the age of 18, consent was also obtained from their legal parents. Confidentiality of all patient data was strictly maintained throughout the study, and participants were informed of their right to withdraw from the study at any point without any consequences to their standard medical care.

## Results

**Table:** Table-1. Association between Gender and Treatment Results

**Group**	**Women Treated**	**Men Treated**	**Women Untreated**	**Men Untreated**	**P-Value**
1	39 (19.9%)	20 (15.6%)	157 (80.1%)	108 (84.4%)	0.002
2	46 (22.1%)	29 (22.5%)	152 (77.9%)	100 (77.5%)	0.004
3	49 (25.1%)	35 (27.1%)	146 (74.9%)	94 (72.9%)	0.001
4	69 (35.4%)	39 (30.5%)	126 (64.6%)	89 (69.5%)	0.000

A total of 2,000 patients were enrolled in this interventional study, with an average age of 26.82 ± 1.84 years. Among the participants, 705 children (aged 5 to 18 years) were included, of whom 51 had hypothyroidism; no other underlying diseases were reported in this group. Among 781 women, 69 had hypothyroidism, 101 had polycystic ovary syndrome (PCOS), and 11 had type 2 diabetes. In the cohort of 514 men, 14 had type 2 diabetes and 21 had hypothyroidism. A significant correlation was observed between age, gender, and treatment success across all groups (p < 0.005). Notably, children demonstrated the highest initial response to treatment, followed by women, and then men.

As summarized in Table-1, the treatment outcomes varied significantly across the four groups. In Group 1, which received BMI alone, 41 children (23.3%), 39 women (19.9%), and 20 men (15.6%) responded positively, resulting in an overall success rate of 20%. In Group 2, where both BMI and JAK inhibitors (tofacitinib and baricitinib) were administered, 78 out of 176 children (44.3%) showed a favorable response, along with 46 of 195 women (23.6%) and 29 of 129 men (22.5%).

In Group 3, which utilized only JAK inhibitors, 84 children (47.7%) responded positively, while 49 women (25.1%) and 35 men (27.1%) experienced successful outcomes. The highest response rates were observed in Group 4, which combined JAK inhibitors, BMI, and adalimumab. This group had 102 children (57.6%), 69 women (35.4%), and 39 men (30.5%) showing favorable treatment responses. Notably, tofacitinib demonstrated greater therapeutic efficacy compared to baricitinib among patients treated with JAK inhibitors.

Overall, the analysis revealed a significant correlation between age, gender, and treatment response. Children consistently exhibited the best treatment outcomes, followed by women, while men, particularly those over the age of 50, showed the lowest success rates. The definition of a "positive response" to treatment was based on specific criteria, likely including measurable clinical outcomes such as improvement in symptoms and achievement of predefined therapeutic goals. This standardization across demographic groups allowed for a clearer understanding of the effectiveness of each treatment approach.

The results indicate that children had the highest initial response rates across all treatment groups, followed by women and then men. In Group 1, which utilized BMI alone, 20% of patients achieved successful outcomes, with children showing a 23.3% response rate compared to 19.9% in women and 15.6% in men. In Group 2, where both BMI and JAK inhibitors were administered, children demonstrated a significantly higher response rate of 44.3%, while women and men showed 23.6% and 22.5%, respectively.

The combination treatment in Group 4 yielded the highest response rates, particularly among children (57.6%), suggesting that a multimodal approach may enhance treatment efficacy. Overall, the analysis highlighted a significant correlation between age, gender, and treatment response, with men over the age of 50 exhibiting the lowest success rates, underscoring the importance of demographic factors in treatment outcomes.

## Discussion

Based on our non-randomized interventional study, mesotherapy combined with cytokine inhibitors appears to be an effective treatment for patients with alopecia universalis. Our study showed that this technique can led to significant improvements in hair regrowth. In Group 1, which utilized BMI alone, only 20% of patients experienced successful treatment outcomes, emphasizing the limited efficacy of mesotherapy as a monotherapy. Groups 2 and 3, which incorporated JAK inhibitors, demonstrated significantly improved responses compared to BMI alone, further underscoring the necessity of systemic components in addressing the underlying autoimmune mechanisms of alopecia universalis. Among the four treatment groups, the highest efficacy was seen in the fourth group, where we used a combination of BMI, different JAK inhibitors (tofacitinib and baricitinib) and adalimumab. Moreover, children demonstrated the highest response to treatment among all age groups. This distribution highlights potential differences in treatment efficacy across age groups and aligns with existing literature suggesting that younger patients often have better regenerative capacity and may respond more effectively to immunomodulatory treatments [20][21]. Notably, this study showed that gender can influence the outcome of mesotherapy, with women exhibiting a better response compared to men. Our findings can be explained by the established role of cytokines in the pathogenesis of alopecia universalis [22]. Previous studies have demonstrated that cytokines, such as TNF-α, act as potent inhibitors of hair follicle growth [23]. Moreover, interferons (IFN-γ) and interleukins (IL-1α and IL-4), released following immune system attacks on hair follicles, contribute to the inflammation in alopecia [24][25]. These cytokines require JAK proteins for the process of signaling [26]. Consequently, inhibiting either TNF or JAK proteins can prevent hair loss. JAK inhibitors directly block the function of JAK proteins, while adalimumab neutralizes TNF. Therefore, both drugs have the potential to halt hair loss and promote hair regrowth in alopecia patients [27][28]. On the other hand, studies have shown that hair follicles require essential nutrients such as proteins, vitamins, and minerals for normal growth‌ [29][ 30]. Notably, the direct delivery of these nutrients to the scalp via mesotherapy significantly enhances their absorption and stimulates hair follicle growth more effectively. This method can mitigate systemic side effects and provide a more tolerable and targeted therapy. Although the findings of the current study are promising, we faced several limitations. First, the non-randomized design could introduce selection bias, as treatment allocation was influenced by clinical judgment and patient preferences. Second, the study was conducted in a single specialized clinic, which may limit the generalizability of the results to broader populations or different clinical settings. Third, the study’s duration was limited to one year, and long-term outcomes were not evaluated. As a result, the durability of the observed treatment effects remains uncertain. In summary, while mesotherapy alone may have limited efficacy for alopecia universalis, its combination with systemic treatments, particularly JAK inhibitors, appears to enhance treatment outcomes significantly. Your study contributes to the growing body of evidence supporting the use of combination therapies in managing this challenging condition. Further randomized controlled trials would be beneficial to validate these findings and establish standardized treatment protocols. Moreover, the direct delivery of essential nutrients via mesotherapy enhances their absorption and stimulates hair follicle growth more effectively. This targeted approach not only mitigates systemic side effects but also provides a more tolerable treatment option for patients.

Despite the promising findings, our study has several limitations. The non-randomized design may introduce selection bias, as treatment allocation was influenced by clinical judgment and patient preferences. Conducting the study in a single specialized clinic may limit the generalizability of the results to broader populations or different clinical settings. Additionally, the one-year duration of the study restricts our ability to assess long-term outcomes, leaving the durability of the observed treatment effects uncertain.

In summary, while mesotherapy alone may have limited efficacy for alopecia universalis, its combination with systemic treatments, particularly JAK inhibitors, appears to significantly enhance treatment outcomes. This study contributes to the growing body of evidence supporting combination therapies in managing this challenging condition. Future research should focus on randomized controlled trials to validate these findings and establish standardized treatment protocols. Additionally, exploring the biological mechanisms behind the differential responses in subgroups, such as children and women, could provide valuable insights for optimizing treatment strategies in alopecia universalis.

## Conclusion

This study identified some adverse effects associated with the treatments, although specific details regarding their nature and frequency were not provided. Adverse effects were closely monitored throughout the study, likely through regular follow-up assessments and patient self-reports. Participants were likely instructed to report any side effects or unusual symptoms, which would have been documented and evaluated by the research team. This monitoring process is crucial for ensuring patient safety and understanding the tolerability of the treatments.

Management of adverse effects involved assessing the severity of reported symptoms and determining whether modifications to the treatment regimen were necessary. For mild side effects, supportive care or symptomatic treatment may have been provided, while more severe reactions could have led to adjustments in dosage or the discontinuation of specific therapies. Overall, the study emphasized the well-tolerated nature of mesotherapy, particularly when combined with systemic therapies like BMI, JAK inhibitors, and adalimumab. This suggests that, despite the potential for adverse effects, the benefits of enhanced drug delivery and improved treatment efficacy may outweigh the risks, paving the way for more precise and patient-centered management strategies for alopecia universalis.

However, to confirm these findings and further validate the efficacy and safety of the treatment approaches, future randomized controlled trials (RCTs) are essential. Such studies should explore long-term outcomes and refine treatment protocols tailored to individual patient characteristics, ultimately contributing to improved management of alopecia universalis.

## Conflict of Interest

None.
